# Combined conventional speech therapy and functional electrical stimulation in acute stroke patients with dyphagia: a randomized controlled trial

**DOI:** 10.1186/s12883-022-02753-8

**Published:** 2022-06-22

**Authors:** Klayne Cunha Matos, Vanessa Fernandes de Oliveira, Paula Luanna Carvalho de Oliveira, Fabíola Aureliano Carvalho, Maria Renata Matos de Mesquita, Camila Gabriella da Silva Queiroz, Levi Mota Marques, Débora Lilian Nascimento Lima, Fernanda Martins Maia Carvalho, Pedro Braga-Neto

**Affiliations:** 1 Speech Therapy Service, Hospital Geral de Fortaleza, Fortaleza, Brazil; 2 Department of Clinical Medicine, Universidade Federal do Ceará, Fortaleza, Brazil; 3 Department of Medicine, Centro Universitário INTA, Sobral, Brazil; 4 Otorhinolaryngology Service, Hospital Geral de Fortaleza, Fortaleza, Brazil; 5 Medical Sciences Post-Graduation Program, Universidade de Fortaleza, Fortaleza, Brazil; 6 Neurology Department, Hospital Geral de Fortaleza, Fortaleza, Brazil; 7 Center of Health Sciences, Universidade Estadual do Ceará, Fortaleza, Brazil

**Keywords:** Dysphagia, Electrical stimulation, Rehabilitation, Speech therapy, Stroke

## Abstract

**Background:**

Stroke is the main cause of oropharyngeal neurogenic dysphagia. Electrostimulation has been used as a therapeutic tool in these cases. However, there are few studies that prove its effectiveness. We evaluated the effect of functional electrostimulation as a complement to conventional speech therapy in patients with dysphagia after a stroke in a stroke unit.

**Methods:**

We performed a clinical, randomized, and controlled trial divided into intervention group (IG) (*n* = 16) and control group (CG) (*n* = 17). All patients were treated with conventional speech therapy, and the IG also was submitted to the functional electrotherapy. Primary outcomes were Functional Oral Ingestion Scale (FOIS) and Swallowing videoendoscopy (FEES). The degree of dysphagia was scored in functional, mild, moderate and severe dysphagia according to FEES procedure. Dysphagia Risk Evaluation Protocol (DREP) was considered a secondary outcome.

**Results:**

There was a significant difference regarding FOIS scores after 5 days of intervention in groups. Both groups also showed a tendency to improve dysphagia levels measured by FEES, although not statistically significant. Improvements on oral feeding was seen in both groups. No significant differences between groups before and after the intervention were detected by DREP scores. Electrical stimulation did not show additional benefits beyond conventional therapy when comparing outcomes between groups.

**Conclusion:**

Conventional speech therapy improved oral ingestion even regardless the use of electrostimulation in a stroke unit.

**Trial registration:**

This research was registered in ClinicalTrials.gov (Identifier: NCT03649295) in 28/08/2018 and in the Brazilian Registry of Clinical Trials (ReBEC) (Register Number: RBR-56QK5J), approval date: 18/12/2018. HGF Ethics Committee Approval Number: N. 2.388.931.

## Introduction

Stroke is the most disabling disease and the first cause of death in adults in Brazil. It is also pointed out as the third leading cause of death in the world [1, 2]. Mortality rate reaches 20% every month and about one third of survivors remain dependent after six months [3].

Dysphagia is a disorder defined as impairment or difficulty in swallowing that can occur in any phase of the swallowing process due to neurological and/or structural causes resulting in anomalous retard in the food bolus transit [4, 5]. Oropharyngeal dysphagia has been previously described as a prevalent symptom in stroke patients, with high associated morbidity and mortality [6] Up to 50% of all stroke patients may have dysphagia [7]. Dysphagia can cause serious complications such as malnutrition, dehydration and aspiration of food or secretions, which can lead to pneumonia [8].

Dysphagic patients after stroke should be assisted as immediately as possible to minimize sequelae and promote their best rehabilitation, in addition to reducing hospital stay [9]. The treatment for dysphagia after acute stroke should be one of the priorities for patient rehabilitation to restore the nutritional status, as to avoid respiratory complication related to aspiration [10]. In the acute phase, patients should ideally be submitted to daily therapy sessions at least 5 days a week [11]. Swallowing abnormalities recover spontaneously in a few patients with stroke [12]. However, symptoms may often persist and should be evaluated in the acute phase of the disease to prevent worsening of symptoms and to improve patient's quality of life [6, 13].

Dysphagia rehabilitation includes several exercises to restore the ability to swallow, improving muscle function and promoting progressive decrease in enteral supplementation as oral diet increases [2]. Isotonic muscle contraction and cryostimulation associated with sour taste and direct therapy using compensation strategies (cleaning maneuvers and Masako’s maneuvers), encompassing the external control of the swallowing process, are some examples of commonly used interventions used by speech therapists worldwide [2]. However, the complexity of swallowing process and the heterogeneity of dysphagia mechanisms make it difficult to precisely determine the real effect of these different treatment techniques on patient outcome [14].

Considered one of the current therapeutic options for oropharyngeal dysphagia, functional electrical stimulation (FES) has been used since 1996 in the United States [15]. This procedure has been approved by the Food and Drug Administration (FDA) in 2001 for the treatment of patients with neurogenic dysphagia aiming at promoting suprahyoid and laryngeal movements and contraction of muscle groups directly involved with swallowing [16]. Therapeutic FES was proposed as a treatment option for oropharyngeal dysphagia, demonstrating benefits for patients after stroke, radiotherapy, dry mouth, tension, and pain, with favorable improvements in vocal quality and swallowing [17].

The efficacy of FES in the treatment of dysphagia after stroke is still controversial. Even though there are a few positive results for the of FES after stroke, its use as routine rehabilitation strategy is still questionable. Moreover, little is known about the specific FES effects on the biomechanical aspects of the pharynx, larynx and on swallowing itself [18].

Crary and Carnaby-Mann (2007) [19] conducted a study to collect large-scale information regarding patterns of practice, outcomes, complications, and perceptions reported by practitioners and associated with electrical stimulation in dysphagia therapy approaches. Practitioners have reported positive uncomplicated clinical results from electrotherapy treatment. However, these professionals did not employ specific criteria for the application of the techniques, did not use standard treatment protocols, and often did not follow up patients beyond the treatment period.

The efficacy of FES in patients with acute stroke in a stroke unit is even more controversial and with scarce literature. The present study aims to point out the benefits of different dysphagia rehabilitation techniques, such as conventional therapy and electrotherapy. It was hypothesized that patients who receive FES added to conventional exercises in a short period of time and in a stroke unit, present a greater improvement in swallowing pattern than patients receiving conventional therapy only. We also questioned whether FES could provide greater safety during the feeding process.

This study aimed to evaluate the effect of a short course functional electrostimulation intervention in a Stroke Unit, as a complement to conventional speech therapy in acute stroke dysphagic patients.

## Methods

We carried out a randomized controlled clinical trial from September 2018 to July 2020 with patients admitted to the Stroke Unit at Hospital Geral de Fortaleza (HGF), a stroke reference tertiary public care hospital in the city of Fortaleza, located in Northeast of Brazil.

### Design

Data collection started when stroke diagnosis was confirmed. Patients were randomly divided into intervention group (IG) and control group (CG) and then submitted to swallowing videoendoscopy (FEES) before starting speech therapy intervention. The initial assessment comprised: Glasgow scale; epidemiological and socio-economic profile; Dysphagia risk evaluation protocol (DREP) and Functional oral intake scale (FOIS). The speech therapist responsible for the initial and final assessments was blind to the study.

According to the Guidelines for the Early Management of Patients with Acute Ischemic Stroke (2019) [20], the choice of an instrumental test may be based on the instrument availability or other considerations (i.e., endoscopic assessment of swallowing, videofluoroscopy, endoscopic assessment of swallowing with sensory test). Our service does not offer the swallowing videofluoroscopy (VFS) exam for dysphagia evaluation. Instead, we perform swallowing videoendoscopy exam which requires trained professionals for such procedure. Endoscopic evaluation is an efficient method to verify the presence/absence of aspiration, to determine the physiological reasons for dysphagia and to guide the treatment plan.

The FEES examination is an accessible and excellent method for the study of swallowing disorders because of the many advantages. It is easy to use and well tolerated. It also allows bedside examinations and it is economical. It has minor risks, and the most likely consequences include discomfort, gagging and/or vomiting, vasovagal syncope, mucosal perforation, adverse reactions to topical anesthetics, and laryngospasm [21].

Although they are not the most frequently used instruments in scientific publications with the objective of assessing the degree of dysphagia, DREP and FOIS scale are usually performed at HGF as the main instruments to assess and determine the degree of dysphagia in patients after stroke. In addition, the hospital only provides the device for FEES evaluation, and it is not possible to choose videofluoroscopy for an objective assessment.

In the IG, conventional speech therapy was performed with electrotherapy, while in the CG speech therapy was performed with the electrodes positioned and the electrotherapy device turned on at intensity 0. Five 20 min sessions were performed, once a day, according to the protocol of functional electrostimulation in dysphagia [18].

**Fig. 1:**
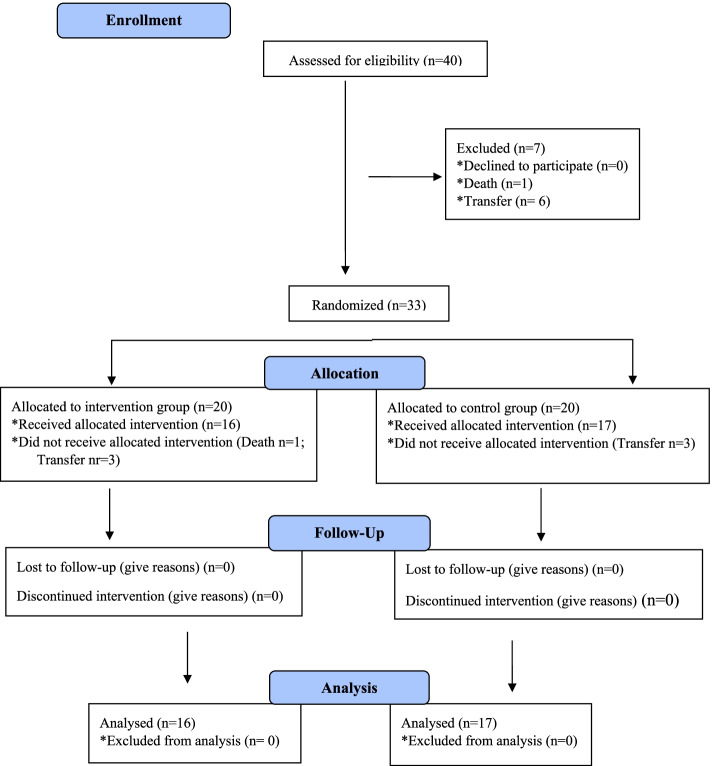
Study design

After 5 sessions, patients were again submitted to FEES, in addition FOIS and DREP were reassessed. Initial and final assessments, FEES, electrotherapy, and conventional speech therapy were performed by two Speech-Language Pathologists. The study design flow chart is shown in Fig. 1. We decided to perform only 5 days of session because the stroke unit of HGF has a high turnover of patients to support a high demand of patients admitted with an acute stroke. Patients are usually transferred to other sectors of the hospital or even other hospitals without the support required to continue the sessions evaluate the outcomes.

### Participants

This is a study with patients in the acute phase of stroke, admitted to the Stroke Unit at Hospital Geral de Fortaleza – (UAVC HGF), where an average of 1000 patients are assisted annually by an interdisciplinary team that includes doctors, nurses, speech therapists, physiotherapists, psychologists, and social workers. As an exploratory study, no formal sample calculation was performed patient’s turnover in the stroke unit was considered, as well as the average monthly number of patients with ischemic stroke admitted [22–24].

The study population included patients with ischemic stroke confirmed by neuroimaging within 24 h of the event, admitted from July 2017 to July 2020 who had oropharyngeal dysphagia as a symptom and required enteral tube feeding. Patients’ age had to be between 40 and 70 years and Glasgow Coma Scale Score should be > 11. Only patients with oropharyngeal dysphagia acquired after stroke were considered. Subjects with degenerative neurological diseases, neoplasia, pacemaker, cochlear implant, feverish state, pregnancy and/or anxiety were excluded.

Patients were divided into two groups: intervention (IG) and placebo control group (CG). Initially, 40 participants were selected. However, during the study, 1 IG patient died and 3 were transferred to a support hospital that did not have structure to perform the necessary procedures to this study. Likewise, 3 patients from CG were transferred. The study was completed with 33 patients, 16 in the intervention group and 17 in the placebo group.

### Ethical approval and consent to participate

Informed consent was obtained from all subjects and/or their legal guardian(s). The ethical determinations of the Resolution no. 466/12 of the National Health Council on research with human beings were followed [25]. All methods were carried out in accordance with relevant guidelines and regulations (declaration of Helsinki) [26]. The study was approved by local Ethics institution (Hospital Geral de Fortaleza).

This research was previously registered in ClinicalTrials.gov (Identifier: NCT03649295) and in the Brazilian Registry of Clinical Trials (ReBEC) (Register Number: RBR-56QK5J).

### Randomization

A block randomization was carried out where there was a random sequence of 10 blocks with 4 participants each, drawn through the *Research Randomizer* program, available at https://www.randomizer.org/. This procedure ensured that the placebo group and the intervention group were balanced in terms of participants’ number.

### Intervention/procedures

Electrostimulation was performed using the Neurodyn Portable Tens/FES technique, with no systemic effect, causing no dependence or undesirable side effects and consists of the application of mild electrical stimulation through electrodes placed on body areas affected by pain, or to activate skeletal muscles and produce contractions. This equipment corresponds to CLASS II type BF for safety and protection and was designed following the existing technical standards for the construction of medical devices (NBR IEC 60,601–1, NBR IEC 60,601–1-2 and NBR IEC 60,601–2-10) Electrotherapy was performed in FES mode, and the following parameters were used: frequency (Hz), electric pulse type (um), intensity (um), TON and TOFF, and ramps to make the contraction as similar as possible to the physiological contraction. A specific FES program was used for patients with oropharyngeal dysphagia consisting of 5 steps [18].

1. Muscle warming up: frequency: 10 Hz; pulse duration: 250 um; TON: 6 s; TOFF: 12 s; stimulus duration: 2 min
2. Type I muscle fibers potentiation: frequency: 30 Hz; pulse duration: 250 um; TON: 5 s; TOFF: 10 s; stimulus duration: 8 min
3. Type II muscle fibers potentiation: frequency: 80 Hz, pulse duration: 300 um; TON: 5 s; TOFF: 10 s; stimulus duration: 8 min
4. Muscle toning: frequency: 30 Hz; pulse duration: 300 um; TON: 5 s; TOFF: 7 s: stimulus duration: 8 min,
5. Relaxation: frequency: 5 Hz; pulse duration: 200 um; TON: off; TOFF: off; stimulus duration: 4 minutes [27].

**Fig. 2:**
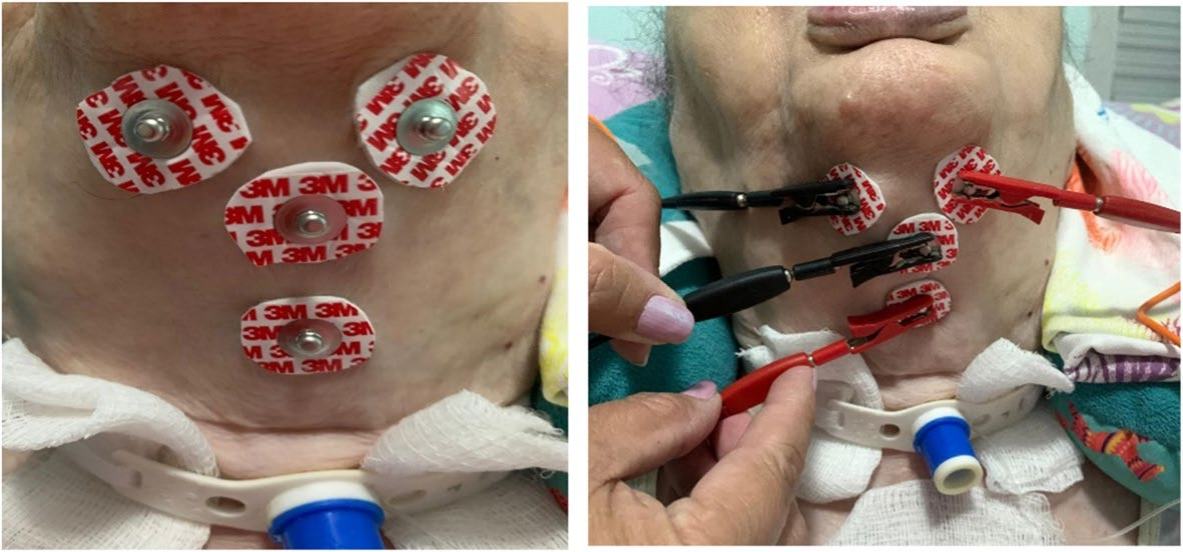
Electrodes position during FES

According to the protocol, one channel of electrodes was placed in the submental region and the other channel over the thyroid cartilage forming a T (Fig. 2). Treatment procedure was explained to the patients, describing the sensations they should expect to happen during stimulation. The current intensity required for the treatment depended on the patient's sensation and, therefore, was applied up to the desired tolerance or level of muscle contraction. Therefore, treatment was started with minimum levels of intensity, increasing carefully until therapeutical levels were achieved and according to the patient's report [18].

Therapeutic session for patients in the intervention group followed these steps: (1) Electrodes were placed in the submental region and on thyroid cartilage, in the supra hyoid region; (2) Device was adjusted to the appropriate initial parameters; (3) The device was switched to “on” mode; (4) Intensity was adjusted from an initial setting; (5) Therapeutic exercises were then performed. Therapeutic session in the patients on the Placebo Group was performed according to the following steps: (1) Electrodes were placed in the submental region and on thyroid cartilage, in the supra hyoid region; (2) Device was adjusted to the appropriate initial parameters; (3) The device was switched to “on” mode and intensity was kept in “0”; (4) Therapeutic exercises were performed.

Initially, patients were submitted to speech therapy evaluation using the aforementioned evaluation instruments (FEES, FOIS, DREP) and sent to the otorhinolaryngology department to perform the initial FEES.

Based on these results, an appropriate therapeutic plan was determined by the two speech therapists responsible for the intervention. Conventional speech therapy consisted of:

- Isotonic exercises (movements that promote greater amplitude and speed): the tongue is taken to the labial commissures maintaining the repetition and rhythm of the movement, sometimes with the professional making resistance with a tongue depressor [28].

- Isometric exercises: to improve strength and resistance of the phono articulatory organs (OFA's) such as tongue, lips and cheeks aiming at rebalancing the strength and amplitude of the movements related to the swallowing process. An example of OFA's isometry: tongue held static in each commissure for a period of 30 s to one minute on each side and on the palate pressing until the stop is ordered [29].

- Exercises to stimulate triggering of the swallowing reflex, relaxation of trismus, and exercises to stimulate intraoral sensitivity were also performed [30].

- Consecutive repetitions of Shaker exercise: head elevations in the supine position, with patients being instructed to lift their heads and advance far enough to be able to observe their toes without lifting their shoulders off bed. This is an exercise proposed to strengthen the suprahyoid muscle [31].

- Gustatory therapy: performed with the therapist's finger in the patient's oral cavity, with a straw, with a cup or with a spoon to rescue the patients' gustatory and olfactory memory [32].

- Mendelsohn's Maneuver: With the fingers—thumb and index—the therapist elevates the patient’s larynx and holds it on top at the time of swallowing, helping in the excursion movement of the larynx and increasing the opening of the upper esophageal sphincter [33].

In active therapy, training was performed with patients sitting with their trunk and neck erect. If passive therapy was required in case of bedridden patients, the bed headboard was raised to at least 45 degrees.

Speech therapy was performed once a day for 5 consecutive days. Exercises were guided and supervised by two speech therapists. Enteral diet was maintained when patient was unable to have a safe oral diet. A mixed diet (oral and enteral) was possible depending on the degree of dysphagia and its symptoms.

Endoscopic evaluation of swallowing as performed with a flexible STORZ model 11,001 RD nasofibroscope coupled to the STORZ TELECAM II video camera and the LG® color monitor. Exams were recorded on a USB device using the PINNACLE video-transfer recorder.

During FEES performance, each patient remained seated and the nasofibroscope was introduced through the nostril without using anesthetic or topical vasoconstrictor. Before beginning the swallowing study, nasal fossae and velopharyngeal closure during phonation and saliva swallowing were observed.

Functional assessment of swallowing started with offering food in a thickened consistency, followed by liquid and solid, tinted with blue inorganic dye to facilitate their visualization during the exam. Liquid consistency food was offered in a glass (40 ml), thickened food was offered in a spoon (5 ml) and solids directly in the patient's oral cavity. Since it was not possible to test all consistencies in patients who had more severe swallowing impairment, food offering was individualized. Laryngeal sensitivity was tested by touching the distal end of the device to the vocal folds (arytenoids and/or ventricular bands), allowing observation of glottal adduction and reflex cough.

Instrumental evaluations were performed and interpreted by the same professionals and allowed visualization of swallowing dynamics, especially in its pharyngeal phase (in the presence of the concomitant pathway between the respiratory and digestive tracts).

### Measures/outcomes

Through a socioeconomic profile form, the following information were collected: gender, age, profession, individual income, and family income. Data on cerebrovascular risk factors were obtained through clinical and laboratory tests performed previously by the patients.

### Results of the following assessment instruments were used as primary outcomes

- Swallowing videoendoscopy (FEES)—It allows studying the physiology of swallowing, assessing the presence of dysphagia and it is also a good method to establish the best diet, to indicate and follow appropriate rehabilitation programs and to plan any other diagnostic investigation. In addition, this endoscopic examination not only provides a static assessment of the upper airway structures, but also allows the assessment of swallowing dynamics [34]. The results were interpreted as follows [4]:

- 1. Functional swallowing: small changes without risk of aspiration or swallowing inefficiency,
- 2. Mild dysphagia: minor oral changes with adequate compensations such as an efficient throat clearing reflex and strong reflex coughing,
- 3. Moderate dysphagia: significant risk of aspiration for one or more consistencies and weak reflex cough,
- 4. Severe dysphagia: silent aspiration, choking difficult to recover, absence of swallowing reflex and impossibility of oral feeding.

- Functional Oral Intake Scale (FOIS)—scale developed to quantify the patient's safe oral intake considering changes in consistency and volume of the diet. It is divided into 7 levels ranging from exclusive enteral diet (level 1) to exclusive oral intake without restrictions [35].

In the initial and final evaluation, the secondary outcome was based on the results of the Dysphagia Risk Evaluation Protocol, which is used to identify changes in the dynamics of the swallowing process, to detect possible risks of bronchoaspiration, and to define the degree of dysphagia [4]. It is divided into three parts, where the first part consists in identification, assessment of oral reflexes, comprehensive and expressive language, aspects of orofacial myofunction. In the second part, a direct swallowing test is performed. Movements of the oral and pharyngeal phases are evaluated from the food intake to the triggering of the swallowing reflex and possible signs and symptoms of dysphagia are checked. The third part refers to the speech therapy diagnosis and the conduct.

We also verified if there was change of dysphagia degree based on the volume of safe oral intake and the possibility of enteral tube removal. The primary outcomes (FOIS and FEES results) determined the safest and the most restrictive level of oral intake. Some variables were analyzed during the swallowing videoendoscopy performance to determine oral feeding safety. Laryngeal penetration was characterized by the presence of dyed food in the laryngeal vestibule (laryngeal face of the epiglottis, aryepiglottic folds, interarytenoid region, vestibular folds, and ventricles, up to the upper face of the vocal folds). Laryngotracheal aspiration was considered present when food was observed in the region located below the vocal folds, subglottic region, and trachea, indicating a moderate or severe degree of dysphagia.

### Statistical analysis

The results analysis was based on the assessment at the beginning of the intervention and after 5 days, as well as on the FEES (initial and final) results for each participant.

Data were analyzed using SPSS version 20.0 with a level of α = 0.05. Descriptive statistics (central tendency and dispersion, absolute and relative frequency) were used to describe the sample characteristics.

Continuous variables (Glasgow and NIHSS) were analyzed intragroup with paired t test, comparing the outcomes before and after the five days of intervention. For intergroup analysis, independent t test was used (the results were presented in differences average with 95% confidence interval). Categorical variables (all other variable in this study) were compared between groups using Pearson's Chi-Square test (χ2).

Results obtained after initial and final evaluations (FEES, FOIS, DREP,) were compared and analyzed statistically, as previously described.

## Results

### Subjects/participants

Final sample consisted of 33 patients. Table 1 describes socioeconomic and demographic data. There were no differences between the intervention and control group. Table 2 describes the clinical profile between intervention and control group. A sedentary lifestyle was the most common risk factor, present in 24 patients (72.7%) and the least incident was dyslipidemia (3 patients or 9%). All patients in both groups had two or more risk factors. There was no statistically significant difference between groups regarding epidemiological profile except for the presence of coronary heart disease, which was statistically more frequent in the control group (*p* = 0.004). Intervention group had also a statistically significant lower level of consciousness measured by Glasgow coma scale.

**Table 1:** Socioeconomic and demographic profile between control and intervention group

Variables		Intervention	Control	P
Age	40 – 50	04 (36.4%)	07 (63.6%)	0.39
	51 – 60	06 (46.2%)	07 (53.8%)	
	61 – 70	06 (66.7%)	03 (33.3%)	
Gender	Male	12 (52.2%)	11 (47.8%)	0.52
	Female	04 (40.0%)	06 (60.0%)	
Family income (minimum wage)	< 2 minimum wage	09 (52.9%)	08 (47.1%)	0.80
	2 – 5 minimum wage	04 (40.0%)	06 (60.0%)	
	> 5 minimum wage	03 (50.0%)	03 (50.0%)

**Table 2:** Clinical profile between control and intervention group

Variables		Intervention	Control	p
Previous Stroke	Yes	05 (55.6%)	04 (44.4%)	0.62
Heredity	Yes	11 (64.7%)	06 (35.3%)	0.08
Diabetes	Yes	06 (54.5%)	05 (45.5%)	0.62
Systemic Arterial Hypertension	Yes	12 (54.5%)	10 (45.5%)	0.32
Dyslipidemia	Yes	03 (100%)	00 (00.0%)	0.10
Alcoholism*	Yes	08 (44.4%)	10 (55.6%)	0.61
Sedentary lifestyle**	Yes	11 (45.8%)	13 (54.2%)	0.62
Obesity	Yes	04 (57.1%)	03 (42.9%)	0.60
Smoking	Yes	07 (46.7%)	08 (53.3%)	0.85
Coronary disease	Yes	01 (10.0%)	09 (90.0%)	0.004^*^
Glasgow	Mean ± SD	14.62 ± 1.02	13.53 ± 1.54	0.02^*^
NIHSS (initial)	Mean ± SD	9.63 ± 5.30	10.53 ± 7.24	0.68
NIHSS (final)	Mean ± SD	8.94 ± 5.53	10.41 ± 7.02	0.51

*NIHSS* National Institute of Health Stroke Scale, *SD* Standard deviation

^*^According to: Diagnostic and Statistical Manual of Mental Disorders (DSM-5) Published by the American Psychiatric Association (APA) [36]

^**^Less than 150–300 min of moderate-intensity aerobic physical activity or at least 75–150 min of vigorous-intensity aerobic physical activity [37]

### Outcomes

#### FEES

Table 3 describes the comparison of FEES measurements between intervention and control group. Groups were homogeneous in the baseline (*p* = 0.80), comprising mostly patients with moderate and severe dysphagia. There was no statistically significant difference between groups after the intervention (*p* = 0,94).

**Table 3:** Comparation of FEES measures between control and intervention group

Variables		Intervention	Control	p
FEES (initial)	Functional Swallowing	00 (00.0%)	01 (100%)	0.80
	Mild Dysphagia	01 (50.0%)	01 (50.0%)	
	Moderate Dysphagia	06 (50.0%)	06 (50.0%)	
	Severe Dysphagia	09 (50.0%)	09 (50.0%)	
FEES (final)	Functional Swallowing	03 (50.0%)	03 (50.0%)	0.94
	Mild Dysphagia	03 (60.0%)	02 (40.0%)	
	Moderate Dysphagia	04 (44.4%)	05 (55.6%)	
	Severe Dysphagia	06 (48.5%)	07 (53.8%)	

FEES: Swallowing videoendoscopy

Although not statically significant, there was a tendency to dysphagia level improvement presented by the patients of both groups after treatment. No significant difference in the intragroup results were observed. At the beginning of the study, no patient presented functional swallowing in the intervention group while 9 patients (56%) had severe dysphagia. After five days of intervention, the number of patients with functional swallowing increased to 3 (18.7%).

Likewise, a tendency to improve swallowing was observed in the placebo group patients after the intervention. Only 1 patient (5.8%) presented functional swallowing during the initial FEES and 9 patients (52.9%) had severe dysphagia. At the final FEES results, it was observed that the number of patients with severe dysphagia decreased to 7 (41.2%) while 3 patients (17.6%) presented functional swallowing. No significant difference was found between groups regarding FEES results.

#### Laryngeal penetration and laryngotracheal aspiration

Table 4 describes laryngeal penetration and laryngotracheal aspiration of liquid and thickened liquid between groups (Table 4). Both groups were homogeneous at the beginning of the study. In the intervention group patients, there was a decrease in laryngeal penetration and laryngotracheal aspiration in the two tested consistencies although no significant differences were observed regarding these variables’ improvement. In the placebo group, there was a decrease in the variables analyzed only in liquid consistency.

**Table 4:** Laryngeal penetration, laryngotracheal aspiration between intervention and control group

Variables		Intervention	Control	p
Initial Laryngeal Penetration (with liquid diet)	Presence	08 (50.0%)	08 (50.0%)	0.86
Final Laryngeal Penetration (with liquid diet)	Presence	05 (50.0%)	05 (50.0%)	0.90
Initial Laryngeal Penetration (with thickened diet)	Presence	08 (66.7%)	04 (33.3%)	0.15
Final Laryngeal Penetration (with thickened diet)	Presence	05 (55.6%)	04 (44.4%)	0.70
Initial Laryngotracheal Aspiration (with liquid diet)	Presence	04 (50.0%)	04 (50.0%)	0.92
Final Laryngotracheal Aspiration (with liquid diet)	Presence	01 (25.0%)	03 (75.0%)	0.60
Initial Laryngotracheal Aspiration (with thickned diet)	Presence	03 (60.0%)	02 (40.0%)	0.65
Final Laryngotracheal Aspiration (with thickned diet)	Presence	01 (25.0%)	03 (75.0%)	0.60

#### FOIS

At the beginning of the study, all patients were on an exclusive enteral diet, scoring level 1 on FOIS. At the end of the study, after conventional speech therapy sessions with or without electrotherapy addition, final FOIS scores were 3.44 ± 2.28 for the IG and 3.18 ± 1.84 for the CG. There was a significant difference in relation to FOIS level improvements after 5 days of sessions in the intervention group (*p* = 0.001) and in the control group (*p* = 0.01). Regarding the intergroup comparison, no significant differences were observed before and after the intervention (*p* = 1.00 and 0.72 respectively).

#### DREP and GAG

Table 5 describes the comparison of initial and final DREP measures between intervention and control group. There were no significant differences between groups before and after the intervention. Groups were homogeneous in the baseline (initial), with no significant differences between the variables (*p* = 0.61). No patient had mild dysphagia or functional swallowing.

**Table 5:** Comparation of Initial and final DREP measures between intervention and control group

Variables		Intervention	Control	P
DREP (initial)	Functional Swallowing	00 (00.0%)	00 (00.0%)	0.61
	Mild Dysphagia	00 (00.0%)	00 (00.0%)	
	Moderate Dysphagia	08 (53.3%)	07 (46.7%)	
	Severe Dysphagia	08 (44.4%)	10 (55.6%)	
DREP (final)	Functional Swallowing	01 (50.0%)	01 (50.0%)	0.57
	Mild Dysphagia	08 (57.1%)	06 (42.9%)	
	Moderate Dysphagia	03 (30.0%)	07 (70.0%)	
	Severe Dysphagia	04 (57.1%)	03 (42.9%)	

*DREP *Risk evaluation protocol for dysphagia

There was a tendency to improvement in the degree of dysphagia among IG patients. After five days of conventional speech therapy associated with functional electrotherapy, 8 patients (50%) progressed to a mild dysphagia degree and it was also observed that 1 patient in this group (6.2%) achieved functional swallowing and the use of enteral diet was waived.

DREP results also showed a tendency to improvement in swallowing pattern on the CG. After five sessions of conventional speech therapy, 1 patient (5.8%) progressed to functional swallowing and the number of patients with mild dysphagia increased to 6 (35.3%).

Table 6 describes GAG, Sialorrhea and Laryngeal elevation measures, data regarding vomiting reflex remained unchanged throughout the study. Two patients in IG decreased sialorrhea while in the CG, this improvement was observed in 5 patients. In the initial laryngeal elevation assessment, IG had 2 patients with adequate movement, and this number increased to 9 patients in the two tested consistencies. In the CG, 3 patients showed appropriate laryngeal excursion movement in the initial evaluation and 10 patients in the final evaluation. None of the analyzed variables showed a significant difference in the intra-group comparison at the end of the intervention.

**Table 6:** Comparisons of GAG, Sialorrhea and Laryngeal elevation measures between groups and controls

Variables		Intervention	Control	p
GAG (initial)	Yes	16 (50.0%)	16 (50.0%)	0.32
GAG (final)	Yes	16 (50.0%)	16 (50.0%)	0.32
Sialorrhea (initial)	Yes	05 (45.5%)	06 (54.5%)	0.80
Sialorrhea (final)	Yes	03 (75.0%)	01 (25.0%)	0.33
Laryngeal elevation (initial) (with thickened diet)	Good	02 (40.0%)	03 (60.0%)	0.68
	Reduced	14 (50.0%)	14 (50.0%)	
Laryngeal elevation (final) (with thickened diet)	Good	09 (33.3%)	10 (66.7%)	0.88
	Reduced	07 (50.0%)	07 (50.0%)	
Laryngeal elevation (initial) (with liquid diet)	Good	02 (40.0%)	03 (60.0%)	0.68
	Reduced	14 (50.0%)	13 (50.0%)	
Laryngeal elevation (final) (with liquid diet)	Good	09 (33.3%)	10 (66.7%)	0.88
	Reduced	07 (50.0%)	07 (50.0%)	

*GAG *Vomiting reflex

#### Nutrition

Table 7 shows the comparison of initial and final feeding routes between groups. Although not statically significant, there was an increase of oral feeding in both groups. At the beginning of the study, 1 IG patient received a mixed diet and all CG patients received exclusive enteral nutrition. After 5 therapy sessions, there were 8 IG patients (50%) with mixed or exclusive oral diet and 6 CG patients (35.3%) also progressed to an oral diet.

**Table 7:** Comparation of Initial and final Feeding routes between intervention and control group

Variables		Intervention	Control	P
Initial feeding route	Nasogastric tube	15 (48.4%)	16 (51.6%)	0.36
	Gastrostomy	00 (00.0%)	01 (100%)	
	Oral	01 (100%)	00 (00.0%)	
Final feeding route	Nasogastric tube	04 (36.4%)	07 (63.6%)	0.58
	Gastrostomy	04 (50.0%)	04 (50.0%)	
	Oral	08 (57.1%)	06 (42.9%)	

## Discussion

The present randomized controlled study investigated if a short-course electrostimulation therapy associated with conventional dysphagia exercises was effective in acute stroke patients in a stroke unit. Sample was composed by a population of stroke patients, composed mostly by male patients. The main finding of our study was a significant improvement in relation to FOIS level in both groups, with no additional benefit on the intervention group in any of the outcomes. Although not statically significant, there was a tendency to improve the dysphagia level in both groups measured by FEES and an increase of oral feeding in both groups.

Oropharyngeal dysphagia has been previously described as a prevalent symptom in stroke patients, with high associated morbidity and mortality. Functional electrical stimulation (FES) has been used as a potentially useful new treatment, although it is still difficult to interpret its effectiveness for the treatment of post-stroke dysphagia [38]. It is important to mention that improvement achieved with FES is seen when this technique is added to conventional therapy.

Similar to our results, previous reports described that improvement in the dysphagia degree is similar in cases where patients receive only conventional therapy and also conventional therapy added to electrotherapy suggesting that conventional exercises can be effective in dysphagia rehabilitation [38–41]. One thing worth mentioning is that most previous studies are related to patients in the subacute phase of stroke and for a longer period of intervention, which may be inaccessible to more vulnerable populations. In a meta-analysis involving 11 randomized clinical trials conducted between 2014 and 2019, the authors confirmed that electrical stimulation helps improving swallowing function in patients post-stroke dysphagia [38].

Our results differ from the results of the study carried out between August 2017 and July 2019 which involved a total of 72 patients with dysphagia after acute stroke divided into two groups, intervention group (electrotherapy) and control group (conventional therapy) [42]. The authors observed significant differences in the swallowing pattern of patients in the intervention group, concluding that electrotherapy added to conventional therapy can be considered an effective technique in the treatment of dysphagia after acute stroke compared to conventional therapy. It should be noted that these patients had daily sessions during four weeks, a total of 20 therapeutic sessions. The number of patients in our study was smaller and they only had 5 intervention sessions, what may explain the difference in the results.

Conventional exercises have advantages such as low cost and can be available if a speech therapist is accessible, especially in Stroke Units worldwide. Electrotherapy technique requires, in addition to a specific device, continuous acquisition of disposable electrodes, making its routine use often improbable in public institutions where access to more expensive materials is restricted, as in the case of some public institutions in Brazil. However, new techniques for dysphagia rehabilitation are necessary, especially regarding non-cooperative patients. Our study did not include patients with Glasgow Coma Scale score under 11, which may be a group that could benefit from FES, and more studies are needed to treat patients with this profile.

Electrical stimulation has been used since 1997 in the United States, when approved by the Food and Drug Administration (FDA), with the purpose of promoting suprahyoid laryngeal movement and to favor the contraction of the muscle groups directly involved in swallowing [43]. Similarly, a study conducted by Nam and coworkers [44] in the United States used the hyolaryngeal complex elevation as a parameter for assessing safe swallowing performance in dysphagic patients after stroke. Suprahyoid stimulation induced an increase in anterior hyoid excursion, and infrahyoid stimulation caused an increase in superior laryngeal elevation. The results showed that electrical stimulation associated with conventional therapy can have advantages and improve the swallowing pattern of dysphagic patients after brain injury. It is relevant to note that the muscles responsible for larynx elevation are the suprahyoid muscles, responsible together for the elevation of the hyoid. Infrahyoid muscles are responsible for lowering the larynx and hyoid bone. These two movements make the laryngeal excursion movement complete, which may explain the results presented in the study cited where the group receiving electrical stimulation applied to the supra and infrahyoid muscles showed greater improvement compared to the group that received stimulus only in the suprahyoid region [39].

Similar results were found in a study carried out to evaluate swallowing in post-stroke patients with dysphagia after four weeks of electrical stimulation of the suprahyoid muscles. The conclusion of this study was that electrical stimulation of the suprahyoid muscles significantly reduced the duration of the oral and pharyngeal phases in patients with post-stroke dysphagia resulting in better swallowing [40].

Another point to be highlighted is that the electrical motor stimulus is more efficient to achieve the contraction of specific muscles, as in the case of the muscles responsible for the laryngeal movement during swallowing. It is naturally possible that this stimulus promotes greater laryngeal elevation if compared to sensory stimulus only, offering greater possibility of recovering adequate metabolic conditions, memory reacquisition, proprioception and improving muscle fibers' atrophy state. The protocol used on our study made a combination of the two stimuli to increase effectiveness of the intervention. Unfortunately, there is no universally accepted FES protocol for dysphagia, including current intensity, frequency, and treatment duration, and it is necessary to optimize electrical stimulation parameters to improve dysphagia treatment.

Laryngeal penetration as well as laryngotracheal aspiration (both with food in liquid and thickened liquid consistencies) were analyzed in this study by performing FEES. In IG patients, there was a decrease in the presence of laryngeal penetration and laryngotracheal aspiration in the two tested consistencies although no significant differences were observed related to these variables’ improvement. In the placebo group, there was a decrease in the variables analyzed only with liquid consistency food. In a study investigating the efficacy and safety of swallowing therapy based on conventional exercises and electrotherapy for rehabilitation of dysphagia after stroke, 53 patients were randomized to groups of swallowing therapy and active electrotherapy, swallowing therapy and placebo electrotherapy or conventional therapy [41]. After treatment, it was shown that conventional therapy with or without electrotherapy was an effective technique to rehabilitate the swallowing pattern of dysphagic patients after stroke [41]. On the present study, the protocol used was for a shorter period and in the acute phase, which may have influenced the results, even though a tendency was observed.

Our results pointed to a significant improvement of FOIS in both groups after the conventional speech therapy sessions with or without electrotherapy addition. Conversely, no significant differences were observed in the intergroup analysis. These results demonstrated that conventional speech therapy, (associated or not with electrotherapy) brings important benefits for the swallowing pattern improvement of patients with dysphagia after stroke. Studies carried out in Taiwan, Czech Republic and in the USA were consistent in demonstrating significant differences in FO IS results in dysphagic patients affected by stroke and treated with conventional therapy associated with electrotherapy support the results obtained in this study [23, 40, 45, 46], which may lead us to attribute this improvement to conventional therapy, although this study was not designed for this purpose.

In accordance with the results of the study mentioned above and the FOIS results in the present study, Lee et al. compared the effect of early treatment using neuromuscular electrical stimulation combined with traditional dysphagia therapy versus isolated traditional dysphagia therapy in 57 patients with acute ischemic stroke with moderate to severe dysphagia. Both groups showed a significant improvement in FO IS after treatment. However, the FOIS score was significantly better 3 and 6 weeks after baseline in the group receiving conventional therapy plus electrostimulation [47]. This study was carried out through 15 rehabilitation sessions and had a follow-up of 3, 6 and 12 weeks, which differs from our study, where electrical stimulation was concentrated on five consecutive days, without follow-up, a factor that may limit the results of the FOIS data collected. A short protocol of FES was used to guarantee access to this technique, since it is available only on a comprehensive care stroke center, which is a reality in most low-income countries.

Huang et al. (2014) [22] have also evaluated electrotherapy use in a prospective study in Taiwan where patients were divided and submitted to conventional therapy, electrotherapy, and combination therapy (traditional and electrotherapy). The results showed significant differences before and after speech therapy intervention in patients who received conventional therapy only and those who received combined therapy, according to FOIS and PAS (*p* = 0.05). There was no significant improvement in the results of patients who received electrotherapy only [22] coinciding and reinforcing the results observed in the present study. Despite the evidence that isolated electrotherapy does not cause additional benefits in dysphagic patients after stroke, this technique is efficient when used in addition to conventional exercises. The variation in symptoms and degrees of dysphagia cases make it difficult to develop an efficient protocol capable of confirming the effectiveness of this technique used in isolation.

The results presented in this study showed that there was no significant difference in the intergroup FOIS levels, despite the significant improvement in both groups analyzed before and after the intervention. Toyama et al. (2014) [24] have also compared the effects of electrotherapy added to conventional treatment in patients with dysphagia after brain injury, using FOIS as a parameter for improving the swallowing pattern in Japan. The experimental group received FES intervention followed by conventional treatment and the control group received conventional treatment without FES. Collected data suggested that electrical stimulation combined with conventional treatment is superior to conventional treatment alone, with significant improvements in all parameters in the experimental group. FOIS mean values ​changed significantly, from 3.8 to 5.2 in the experimental group (*p* < 0.05) and from 4.0 to 4.6 in the control group (*p*< 0.05). However, there was no difference between the two groups in the FOIS results evaluation, such as the ones obtained through the present study [24].

GAG and the swallowing reflex accompanied by the laryngeal excursion or laryngeal elevation movements are oral defense reflexes assessed by DREP. Dysphagia Risk Evaluation Protocol (DREP) data was considered secondary outcome of the present study. There was a decrease in the number of patients with sialorrhea at the end of the study as seen in the study conducted by Li (2015) [48] in China, where 135 patients were divided into 3 groups: electrotherapy group, traditional swallowing therapy group and electrotherapy plus conventional therapy group. After the treatment, the swallowing patterns in each group significantly increased, indicating that electrotherapy associated with conventional therapy and techniques used separately are beneficial to the swallowing safety of dysphagic patients after stroke.

The small sample size was the main limitation of this study. Multicentric studies with larger samples must be carried out including homogeneous populations to obtain more consistent results. Other limiting factors were the availability of only one FEES device, delay in data collection and absence of the Videofluoroscopic Swallow Study (gold standard for swallowing assessment), despite the fact that the FESS presented good sensitivity with high overall values (≥ 80%) and good specificity in relation to the posterior leakage of semi-solids (84.4%) and liquids (86.7%) [49].

Other limiting factors were the availability of only one FEES device, delaying data collection and the absence of Videofluoroscopic Swallowing Study (gold standard for swallowing evaluation). We also did not use a validated scale to evaluate FEES. Furthermore, the site of the stroke lesion was also not evaluated and patients with bulbar or pseudobulbar affection could have a worse dysphagia prognosis. Finally, the study did not include the use of any patient-reported outcome measures as a secondary outcome measurement and patients were not followed after the final evaluation. Despite this, this was the first clinical trial conducted in Brazil with the objective of analyzing the benefits of a short-course functional electrotherapy added to conventional therapy at a Stroke Unit in dysphagic acute ischemic stroke patients. Early intervention of dysphagia patients after stroke could improve oral feeding and a safer oral intake. We believe that early treatment of dysphagic patients after stroke regardless the use of FES could promote a faster rehabilitation and reduce complications like pneumonia.

Through the results presented in this study and supported, including, by studies carried out worldwide on the health and rehabilitation of patients affected by stroke with dysphagia, we see the need to implement measures to improve care for these patients, such as: application of a more efficient routine protocol, inclusion of speech therapists in a public clinical setting, facilitating the continuation of the rehabilitation process of patients with dysphagia affected by stroke both in the acute and in the chronic phases, the need of more studies with larger samples to present more solid and statistically significant results.

## Conclusion

Based on the results of the present study, there was a significant difference in terms of the improvement in the FOIS level of patients in both the intervention group and the placebo group, with no significant difference between groups.

The use of electrical stimulation protocol for a 5 day-period on the acute phase of ischemic stroke apparently did not generate additional benefits beyond conventional therapy, with improvements in variables analyzed in both groups. Conventional speech therapy is possibly responsible for improvements seen in both stroke groups, even when applied isolated.

Despite the small sample size, speech therapy performed with conventional exercises with or without electrotherapy is an important tool to be used in the process of rehabilitation of the swallowing pattern of dysphagic stroke patients using an enteral diet.

## Data Availability

The datasets generated and/or analyzed during the current study are not publicly available due to authors decisions but are available from the corresponding author on reasonable request.

## References

[1] De Carvalho JJF, Alves MB, Viana GÁA (2011). Stroke epidemiology, patterns of management, and outcomes in Fortaleza, Brazil: A hospital-based multicenter prospective study. Stroke.

[2] Drozdz D, Mancopes R, Silva AMT, Reppold C (2014). Analysis of the level of Dysphagia, anxiety, and nutritional status before and after speech therapy in patients with stroke. Int Arch Otorhinolaryngol.

[3] Fukujima MM. Distúrbios neurológicos adquiridos: linguagem e cognição. In: Ortis KZ, ed. Second. Manole; 2010:34–46.

[4] Padovani AR, Moraes DP, Mangili LD, de Andrade CRF (2007). Dysphagia Risk evaluation protocol (PARD). Revista da Sociedade Brasileira de Fonoaudiologia.

[5] Azer SA, Kshirsagar RK (2021). Dysphagia.

[6] Smithard DG, Neill PAO, England RE, et al. The natural history of dysphagia following a stroke. 2014;193:188–93. 10.1007/PL00009535. 10.1007/PL00009535 9294937

[7] Smithard DG, Smeeton NC, Wolfe CDA. Long-term outcome after stroke: does dysphagia matter? Age Ageing. Published online 2007. 10.1093/ageing/afl149. 10.1093/ageing/afl149 17172601

[8] Mendes FS, Tchakmakian LA. Quality of life and interdisciplinarity: the necessity of home care programs in the prevention of complications in old people with dysphagia. O mundo da saúde. 2009;33. 10.15343/0104-7809.200933.3.8.

[9] Andrews AW, Li D, Freburger JK (2015). Association of rehabilitation intensity for stroke and risk of hospital readmission. Phys Ther.

[10] Smithard DG, Smeeton NC, Wolfe CDA. Long-term outcome after stroke: does dysphagia matter? Age Ageing. Published online 2007. 10.1093/ageing/afl149. 10.1093/ageing/afl149 17172601

[11] Archer SK, Wellwood I, Smith CH, Newham DJ (2013). Dysphagia therapy in stroke: a survey of speech and language therapists. Int J Lang Commun Disord.

[12] Arreola V, Vilardell N, Ortega O (2019). Natural history of swallow function during the three-month period after stroke. Geriatrics.

[13] Arreola V, Vilardell N, Ortega O (2019). Natural history of swallow function during the three-month period after stroke. Geriatrics.

[14] Archer SK, Wellwood I, Smith CH, Newham DJ (2013). Dysphagia therapy in stroke: a survey of speech and language therapists. Int J Lang Commun Disord.

[15] Freed M, Christian M, Beytas E, Tucker H, Kotton B (1996). Electrical stimulation of the neck: a new effective treatment for dysphagia. Dyphagia..

[16] Shaw GY, Sechtem PR, Searl J, Keller K, Rawi TA, Dowdy E (2007). Transcutaneous neuromuscular electrical stimulation (VitalStim) curative therapy for severe dysphagia: myth or reality?. Annals of Otology, Rhinology & Laryngology.

[17] Boswell NS (1989). Neuroelectric therapy eliminates xerostomia during radiotherapy – A case history. Med Electron.

[18] Guimarães BT, Guimarães MSM (2013). Eletroestimulação Funcional Em Disfagia Orofaríngea. (Editorial P, ed.).

[19] Crary MA, Carnaby-Mann GD, Faunce A (2007). Electrical stimulation therapy for dysphagia: Descriptive results of two surveys. Dysphagia.

[20] Powers WJ, Rabinstein AA, Ackerson T (2019). Guidelines for the early management of patients with acute ischemic stroke: 2019 update to the 2018 guidelines for the early management of acute ischemic stroke a guideline for healthcare professionals from the American Heart Association/American Stroke Association. Stroke.

[21] Nacci A, Ursino F, Vela R, Matteucci F, Mallardi V (2008). Fiberoptic endoscopic evaluation of swallowing (FEES): proposal for informed consent. Acta otorhinolaryngol Ital.

[22] Huang KL, Liu TY, Huang YC, Leong CP, Lin WC, Pong YP (2014). Functional outcome in acute stroke patients with oropharyngeal Dysphagia after swallowing therapy. Journal stroke cerebrovasc dis.

[23] Sun SF, Hsu CW, Lin HS (2013). Combined neuromuscular electrical stimulation (NMES) with fiberoptic endoscopic evaluation of swallowing (FEES) and traditional swallowing rehabilitation in the treatment of stroke-related dysphagia. Dysphagia.

[24] Toyama K, Matsumoto S, Kurasawa M, et al. Novel Neuromuscular Electrical Stimulation System for Treatment of Dysphagia after Brain Injury. Neurol Med Chir (Tokyo). 2014;54. 10.2176/nmc.oa.2013-0341. 10.2176/nmc.oa.2013-0341 PMC4533457 24670314

[25] Brasil. Caderno de Atenção Domiciliar. 1st ed.; 2012.

[26] Williams JR (2008). The Declaration of Helsinki and public health. Bull World Health Organ.

[27] Guimarães BT, Guimarães MSM (2013). Eletroestimulação Funcional Em Disfagia Orofaríngea. (Editorial P, ed.).

[28] Lazarus C (2006). Tongue stength and exercise in healthy individuals and in head and neck cancer patients. Semin Speech Lang.

[29] Todd JT, Lintzenich CR, Butler SG. Isometric and swallowing tongue strength in healthy adults. In: Laryngoscope. Vol 123. 2013:2469–2473. 10.1002/lary.23852. 10.1002/lary.23852 23918664

[30] Especialização em F, Vasconcelos Pereira NA, Rodrigues Motta A, Cristina Vicente LC. Reflexo Da Deglutição: Análise Sobre Eficiência de Diferentes Estímulos Em Jovens Sadios*** Swallowing Reflex: Analysis of the Efficiency of Different Stimuli on Healthy Young Individuals; 2008. 10.1590/s0104-56872008000300004 18852962

[31] Choi JB, Shim SH, Yang JE, Kim HD, Lee DH, Park JS (2017). Effects of Shaker exercise in stroke survivors with oropharyngeal dysphagia. NeuroRehabilitation.

[32] Santoro P, e Silva IL, Cardoso F, Dias E, Beresford H. Evaluation of the effectiveness of a phonoaudiology program for the rehabilitation of dysphagia in the elderly. Archives of Gerontology and Geriatrics. 2011;53(1). 10.1016/j.archger.2010.10.026. 10.1016/j.archger.2010.10.026 21093069

[33] McCullough GH (2014). Effects of the Mendelsohn Maneuver on extent of hyoid movement and UES opening post-stroke. Dysphagia.

[34] Nacci A, Ursino F, Vela R, Matteucci F, Mallardi V (2008). Fiberoptic endoscopic evaluation of swallowing (FEES): proposal for informed consent. Acta otorhinolaryngol Ital.

[35] Crary MA, Mann GD, Groher ME. Initial psychometric assessment of a functional oral intake scale for dysphagia in stroke patients. 2005;86:1516–20. 10.1016/j.apmr.2004.11.049. 10.1016/j.apmr.2004.11.049 16084801

[36] American Psychiatric Association. Diagnostic and Statistical Manual of Mental Disorders. American Psychiatric Association; 2013. 10.1176/appi.books.9780890425596.

[37] WHO (2019). WHO Guidelines on Physical Activity, Sedentary Behaviour.

[38] Alamer A, Melese H, Nigussie F (2020). Effectiveness of neuromuscular electrical stimulation on post-stroke dysphagia: A systematic review of randomized controlled trials. Clin Interv Aging.

[39] Nam HS, Beom J, Oh BM, Han T. Kinematic Effects of Hyolaryngeal Electrical Stimulation Therapy on Hyoid Excursion and Laryngeal Elevation. Dysphagia. 2013;28. 10.1007/s00455-013-9465-x. 10.1007/s00455-013-9465-x 23605128

[40] Konecny P, Elfmark M (2018). Electrical stimulation of hyoid muscles in post-stroke dysphagia. Biomedical Papers.

[41] Carnaby GD, LaGorio L, Silliman S, Crary M (2020). Exercise-based swallowing intervention (McNeill Dysphagia Therapy) with adjunctive NMES to treat dysphagia post-stroke: A double-blind placebo-controlled trial. J Oral Rehabil.

[42] Liang Y, Lin J, Wang H, et al. Evaluating the efficacy of vitalstim electrical stimu- lation combined with swallowing function training for treating dysphagia following an acute stroke. Published online 2021.10.6061/clinics/2021/e3069. 10.6061/clinics/2021/e3069 PMC8552953 34755758

[43] Kl RICE (2012). Neuromuscular Electrical Stimulation in the Early Intervention Population: A Series of Five Case Studies. Internet J Allied Health Sci Pract.

[44] Nam HS, Beom J, Oh BM, Han T. Kinematic Effects of Hyolaryngeal Electrical Stimulation Therapy on Hyoid Excursion and Laryngeal Elevation. Dysphagia. 2013;28. 10.1007/s00455-013-9465-x. 10.1007/s00455-013-9465-x 23605128

[45] Huang KL, Liu TY, Huang YC, Leong CP, Lin WC, Pong YP (2014). Functional outcome in acute stroke patients with oropharyngeal Dysphagia after swallowing therapy. J stroke cerebrovasc dis official J Nat Stroke Association.

[46] Kushner DS, Peters K, Eroglu ST, Perless-Carroll M, Johnson-Greene D (2013). Neuromuscular electrical stimulation efficacy in acute stroke feeding tube-dependent dysphagia during inpatient rehabilitation. Am J Phys Med Rehabil.

[47] Lee KW, Kim SB, Lee JH, Lee SJ, Ri JW, Park JG (2014). The effect of early neuromuscular electrical stimulation therapy in acute/subacute ischemic stroke patients with dysphagia. Ann Rehabil Med.

[48] Li L, Li Y, Huang R, Yin J, Shen Y, Shi J (2015). The value of adding transcutaneous neuromuscular electrical stimulation (VitalStim) to traditional therapy for post-stroke dysphagia: a randomized controlled trial. Eur J Phys Rehabil Med.

[49] Fattori B, Giusti P, Mancini V (2016). Acta Otorhinolaryngologica Italica. Acta Otorhinolaryngol Ital.

